# Das zitternde Herz des Monarchen – Hintergründe zum Herzleiden von Kaiser Maximilian II

**DOI:** 10.1007/s10354-023-01022-8

**Published:** 2023-10-06

**Authors:** Tobias Heusinger, Michael Stolberg

**Affiliations:** 1 Medizinische Fakultät, Universität Würzburg, Josef-Schneider-Str. 2, 97080 Würzburg, Deutschland; 2 Institut für Geschichte der Medizin, Universität Würzburg, Oberer Neubergweg 10a, 97074 Würzburg, Deutschland

**Keywords:** Medizingeschichte, Habsburger, Maximilian II, Leibärzte, Herzzittern, History of medicine, Habsburgs, Maximilian II, Personal physicians, Heart trembling

## Abstract

Es ist in der Geschichtsforschung seit Langem bekannt, dass der Habsburger Kaiser Maximilian II (1527–1576) zeitlebens unter Herzbeschwerden litt. Zahlreiche Biografen erwähnen diesen Umstand. Seine Krankengeschichte und selbst die Ergebnisse der Obduktion seiner Leiche sind überliefert. Nur unzureichend untersucht ist dagegen, wie Maximilians Ärzte dessen häufig als „Herzzittern“, „tremor cordis“ bezeichnetes Herzleiden erklärten, welche Ursachen und Auslöser sie für die Beschwerden verantwortlich machten, im Allgemeinen und im konkreten Fall ihres berühmten Patienten. Der vorliegende Beitrag geht diese Fragen an, primär anhand eines ausführlichen Konsils des kaiserlichen Leibarztes Andrea Gallo aus dem Jahr 1555. Dieses bislang von der Forschung ignorierte Konsil fasst zunächst das damalige „Lehrbuchwissen“ zum Herzzittern zusammen. Im Anschluss daran wendet es sich dem Fall Maximilians zu und eröffnet dabei nicht zuletzt aufschlussreiche Einblicke in dessen seelische Befindlichkeit.

## Abstract (en)

It has long been known in historical research that the Habsburg Emperor Maximilian II (1527–1576) suffered from heart complaints throughout his life. Numerous biographers mention this fact. His medical history and even the results of the autopsy of his body have been handed down; however, it has not been sufficiently investigated how Maximilian’s physicians explained his heart condition, often referred to as “tremor cordis”, and what causes and triggers they held responsible for this complaint in general and in the specific case of their famous patient. This article addresses these questions, primarily on the basis of a detailed consultation by the imperial personal physician Andrea Gallo, dating from 1555. Gallo’s consilium, which has been ignored by scholares so far, first summarizes the state of knowledge on heart tremors at that time. It then turns to Maximilian’s case and provides revealing insights into his mental state.

„Während er von jenem Herzzittern befallen wird, übermannt ihn ein Mangel an Lebenskraft, er leidet unter Atemnot und Schluckbeschwerden und stößt schmerzhafte Seufzer aus, nach derartigem Herzzittern nimmt er wahr, dass seine Körperkräfte geschwächt sind. Es schmerzen alle Extremitäten, der Puls erscheint langsam anschwellend, selten, klein und aussetzend.“ [1]

„Manchmal wird er auch bewusstlos.“ [2]

Diese Zeilen entstammen einem etwa 200 Seiten umfassenden, in lateinischer Sprache verfassten Konsil von Andrea Gallo aus dem Jahr 1555. Die Beschreibung bezieht sich auf niemand Geringeren als den berühmten Habsburger Kaiser Maximilian II. Dieser litt also unter einem „Herzzittern“, einem „tremor cordis“, das von diversen weiteren Beschwerden begleitet war. In seinem Konsil setzt sich Gallo eingehend mit dem Krankheitsgeschehen auseinander, zunächst in einer ausführlichen Diskussion des zeitgenössischen Lehrbuchwissens über das „Herzzittern“ im Allgemeinen und anschließend in Anwendung dieses Wissens auf Maximilians Leiden. Den Abschluss bilden ausführliche therapeutische Empfehlungen.

Das Konsil eröffnet demnach Einblicke in die konkrete ärztliche Deutung frühneuzeitlicher Herzleiden, über welche die Literatur bisher nur wenig Auskunft gibt: Siraisi [3] und Nutton [4], die sich in ihren Studien mit der Medizin der Renaissance auseinandersetzen, betrachten zwar allgemeine Theorien, Gemeinsamkeiten oder Neuerungen der damaligen Zeit. Nutton nimmt dabei etwa die medizinisch-ärztliche Kommunikation oder kontagiöse Erkrankungen und deren damalige Deutung in den Blick. Jedoch fehlen bei den beispielhaft herausgegriffenen Autoren, wie in den meisten Studien zur damaligen Zeit, Hintergründe zur ärztlichen Auslegung von Herzleiden gänzlich.

Stolberg stellt allgemein fest, dass große Werke, Entdeckungen und Theorien der führenden Autoritäten in der Medizin der Renaissance recht gut untersucht sind, aber nur wenig Wissen über die damalige Deutung von Krankheiten in der konkreten ärztlichen Praxis existiert [5].

Auch wenn er sich primär auf das Mittelalter bezieht, liefert Demaitre [6] einige der wenigen verfügbaren Hinweise zur Deutung von Herzleiden: Er berichtet, dass Ärzte verschiedene Zustände wie „cardiaca passio“ oder „tremor cordis“ seinerzeit vielfach nicht voneinander abgrenzten und synonym gebrauchten. Sie hätten Herzleiden häufig mit Fehlzuständen benachbarter Organe in Verbindung gebracht. Zudem spielten in der Genese unter anderem ein im Herzen vorliegendes Ungleichgewicht, erstickende Dämpfe und auch emotionale Affektionen eine Rolle. Diese allgemeinen Konzepte werden aber – anders als im vorliegenden Konsil von Gallo – weder näher spezifiziert noch in einem konkreten Fallbeispiel angewandt.

Wer sich nun Gallos Ausführungen in angemessener Weise nähern will, muss sich auf die uns heute weitgehend fremd gewordene Welt der frühneuzeitlichen Medizin einlassen. Es kann dabei nicht das primäre Ziel sein, rückblickend eine Diagnose in den Begriffen der modernen Medizin zu stellen, beispielsweise einer Herzrhythmusstörung. Eine solche retrospektive Deutung wäre nicht nur zwangsläufig spekulativ. Sie droht auch, einem angemessenen historischen Verständnis im Wege zu stehen, denn das „Herzzittern“ wurde damals im Rahmen der herrschenden medizinischen Theorien ganz anders gedeutet als heute, und auch das Körperverständnis und die Körperwahrnehmung von medizinischen Laien – wie Maximilian – unterschieden sich grundlegend von den heutigen ([7]; Abb. 1).

**Abb. 1:**
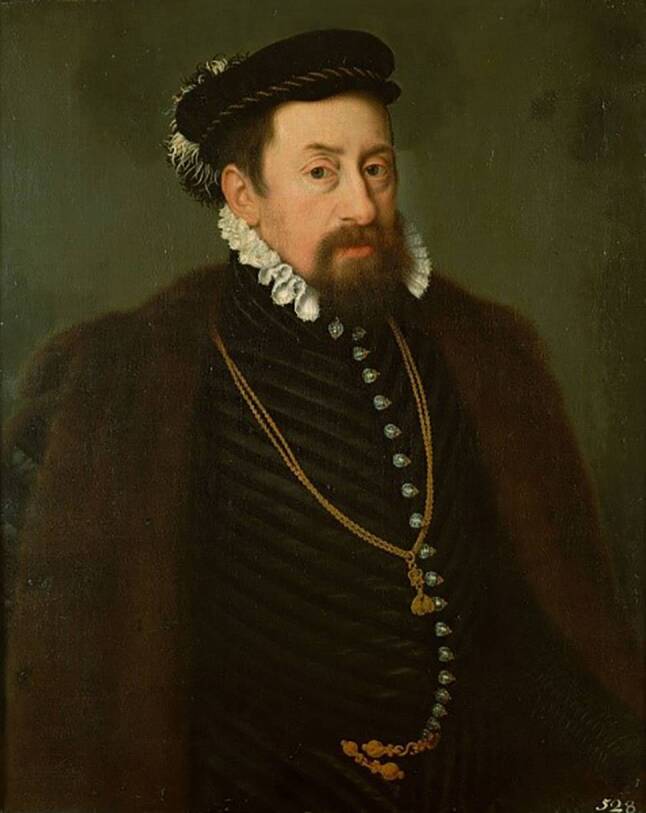
Portrait von Kaiser Maximilian II durch Nicolas Neufchâtel. (Abdruck mit freundlicher Genehmigung des KHM-Museumsverband, © KHM-Museumsverband)

Eine wesentliche Aufgabe frühneuzeitlicher ärztlicher Konsilien war zunächst die Bestimmung der Ursachen und die Identifizierung prädisponierender Faktoren. Mit Blick auf den Kaiser kommt Gallo zu dem Schluss, dass Maximilian an einer kalten *intemperies *des Herzens leidet. Unter der *intemperies *eines Organs verstand man eine Abweichung von einer ausgeglichenen, gesunden Mischung der vier primären, elementaren Qualitäten – kalt, warm, trocken und feucht. In Maximilians Herz ist diese gesunde Mischung durch ein Übermaß an Kälte gestört. Gallo leitet dies aus dem kleinen, langsam anschwellenden und langsamen [8] Puls und aus verschiedenen Charaktereigenschaften des Kaisers ab – das Gefühlsleben und die Affekte wurden damals primär im Herzen verortet. Bezeichnend sind beispielsweise Maximilians Furchtsamkeit, seine Sanftmut oder seine Frömmigkeit [9]. Die schwache Atmung des Kaisers weist in die gleiche Richtung, denn nach damaliger Lehre hatte die Atmung vor allem die Funktion, das Herz zu kühlen. War dieses schon von sich aus eher kühl, genügte eine geringere Atemtätigkeit.

Als Auslöser für das Herzzittern (wie auch für andere Krankheiten) kommen Gallo zufolge unterschiedliche äußerliche und innerliche Einflüsse infrage. Manche von ihnen tragen zur Entstehung des Herzzitterns bei, indem sie das Herz schwächen, andere indem sie diesem einen „Schaden einprägen“ [10]. Zu den äußeren, herzschwächenden Ursachen zählt Gallo im Fall des Kaisers insbesondere starke Affekte und körperliche Belastungen wie Schlafmangel und den Krieg gegen die Franzosen. Auch Maximilians Reisen nach Spanien und die damit verbundenen Strapazen bei stark wechselhaft warmem und kaltem Klima mögen sein Herz geschwächt haben.

Eine Abkühlung und Schwächung des Herzens erklärte freilich noch nicht, warum Maximilians Herzzittern anfallsweise auftrat. Es galt somit zudem, die Einflüsse zu identifizieren, die das Zittern seines kalten und geschwächten Herzens periodisch auslösten. An dieser Stelle bringt Gallo den Einfluss anderer Organe ins Spiel, die das Geschehen im Herzen beeinflussen. Die damaligen Ärzte sprachen in diesem Zusammenhang von einem „Konsensus“ oder einer „Sympathie“ der Organe untereinander.

Der Kaiser hat nach Gallos Beobachtung manchmal Hämorrhoidalblutungen. Diese deuteten im Rahmen der zeitgenössischen Krankheitslehre auf eine „Entzündung“ der Mesenterialgefäße hin, die das Blut „verbrannte“, sodass dieses – schwärzliche – Blut teilweise über die Hämorrhoiden ausgeschieden wurde. Die Mesenterialvenen, so glaubte man, nahmen Speisen aus dem Darm auf und führten sie der Leber zu, wo die Nahrung dann zu nahrhaftem Blut „verkocht“ wurde. Wenn die Mesenterialgefäße entzündet waren, beeinträchtigte dies die Erzeugung guten Bluts in der Leber und damit die Ernährung der einzelnen Organe und nicht zuletzt die des Herzens. Vor allem aber führte die „Entzündung“ dazu, dass zu manchen Zeiten heißer Dampf oder Rauch von den Mesenterialgefäßen zum Herzen aufsteigen konnte und dort aufgrund von dessen Schwächung und seiner *intemperies* anfallsweises Herzzittern hervorrief. Für die zeitgenössischen Mediziner war eine „Entzündung“ nämlich ganz buchstäblich durch starke Hitzeentwicklung gekennzeichnet, bei der das Blut und andere Stoffe unter Rauchentwicklung verbrennen konnten. Dieser Zustand enormer Hitze, der, wenngleich Wärme auch heute eines der vier klassischen Kardinalsymptome von Entzündung ist, anders als eine „Entzündung im modernen Sinn“ interpretiert werden muss, konnte dann über den genannten Mechanismus anfallsweises Herzzittern verursachen.

Der Vergleich von Gallos Ansichten zu den Ursachen von Maximilians Herzzittern mit den mündlichen Äußerungen des berühmten Mediziners Girolamo Fracastoro (1478–1553) zum gleichen Fall lässt erkennen, dass sich Gallo hier eines anerkannten Erklärungsmodells bediente. Fracastoros Ausführungen wurden von einem Zuhörer – wahrscheinlich einem seiner Paduaner Studenten – festgehalten. Ganz ähnlich wie Gallo stellt Fracastoro fest, dass Maximilian an „häufigem Herzzittern und Palpitationen leidet, welche sich allmählich verschlimmern und schließlich zu Ohnmachten führen“ [11]. Der Puls ist häufig, ungeordnet und oft unterbrochen. Fracastoro kann zwar keine krampfhaften Bewegungen (wie bei der Epilepsie) beobachten, neben weiteren Symptomen sei aber oft Blut aus den Hämorrhoiden geflossen. Dazu verweist er auf Maximilians Kummer und Traurigkeit.

Als mögliche, dem Herzzittern zugrunde liegende Ursachen nennt Fracastoro seelische Umstände wie große Schmerzen oder starke Angst. Zudem diagnostiziert er aus Maximilians Ausscheidungen das Vorliegen zähflüssiger und roher Körpersäfte, welche in ihrer Qualität und Quantität stark widernatürlich verändert sind, ja, „Gifte imitieren“ [12]. Man ging damals davon aus, dass rohe und krankhaft veränderte Körpersäfte, ähnlich wie bei Fäulnisvorgängen, Dämpfe bzw. Rauch absondern konnten, welche sich wiederum im Körper verteilten. Diesen Mechanismus vermutet Fracastoro bei Maximilian und nimmt an, dass besagter Rauch leicht zum Herzen und zu den benachbarten Körperteilen gelangen kann und in weiterer Folge Herzzittern und Bewusstlosigkeit verursacht. Er kann sich aber auch weiter ausbreiten und etwa bei „Verstopfung“ der Nerven Albträume auslösen.

Ähnlich wie Gallo geht Fracastoro dabei von prädisponierenden Momenten in Maximilians Herz aus, konkret von einer schlechten *complexio*, also einer ungünstigen Mischung der Qualitäten im Herzen, sowie einem Defizit an *spiritus vitalis*, an Lebensgeist. Der heiße *spiritus vitalis* hatte nach alter Lehre seinen Ort im Herzen. Er breitete sich von dort aus über die Arterien in die übrigen Körperteile aus und versorgte sie mit seiner belebenden Wärme. Ein Mangel an *spiritus *konnte unterschiedliche Ursachen haben wie lange Krankheiten, große Anstrengungen, Schlaflosigkeit, seelische Affekte und übermäßigen Beischlaf [13].

Gallos Empfehlungen zur arzneilichen Behandlung von Maximilians Herzzittern zielen folgerichtig zunächst einmal darauf, die kalte *intemperies* des Herzens zu korrigieren durch wärmende Arzneien wie Fenchel, Anis, Johanniskraut und Pfeffer, wenn nötig verbunden mit der Gabe von kühlenden Substanzen, um wiederum eine zu starke Erhitzung zu verhindern. Dazu kommen herzstärkende Mittel. Von dem damals sehr häufig angewandten Aderlass rät Gallo dagegen ab. In Maximilians Herz selbst findet sich, anders als bei den meisten anderen Krankheiten, keine widernatürliche, krankhafte Feuchtigkeit, von der man das Herz und den Körper insgesamt befreien müsse. Gallo warnt im Gegenteil vor den Gefahren des Aderlasses, weil der Verlust des warmen Bluts die kalte *intemperies* sogar noch verschlimmern würde.

Bekanntlich nehmen Ärzte heute bei der Therapie von Krankheiten – neben der Verabreichung von Medikamenten – immer stärker auch die Lebensweise des Patienten in den Blick und fordern nicht selten auch eine sog. Lifestyle-Modifikation von ihren Patienten ein. Diätetischen Empfehlungen kam jedoch in der frühneuzeitlichen ärztlichen Krankheitsbehandlung neben der Verabreichung von Medikation noch weit größere Bedeutung zu als heute. In seinem Konsil gibt Gallo detaillierte Anweisungen. Sie eröffnen zusammen mit seinen Überlegungen zu den Ursachen von Maximilians Herzzittern zugleich aufschlussreiche Einblicke in dessen bisherige Lebensweise.

Wie erwähnt, macht Gallo mangelnden Schlaf und die Anstrengungen des Kriegs gegen die Franzosen mitverantwortlich für Maximilians Herzzittern. Er empfiehlt seinem Patienten, in Zukunft zwölf Stunden pro Tag zu schlafen und zwölf Stunden wach zu sein. Dies ist ungewöhnlich, da die Ärzte meist sieben Stunden Schlaf empfahlen. Gallo scheint seinem Patienten an dieser Stelle jedoch nicht exzessiven Schlaf anraten zu wollen, stattdessen geht es ihm um die Ausgewogenheit von Schlafen und Wachen. Auch wenn die Darstellung im Konsil an dieser Stelle nicht ganz zweifelsfrei nachvollziehbar ist, begründet Gallo seine Empfehlung und die Beobachtung, dass Maximilians Herzzittern nur manchmal beim Schlafen aufhört, wie folgt: Das Schlafen trägt dazu bei, dass rohe Körpersäfte verkocht werden – ein positiver Effekt, da die Anhäufung roher Säfte im Körper, wie bereits erwähnt, mit negativen Folgen einhergeht. Allerdings verhindert der Schlaf die gesundheitsfördernde „Austreibung“ und Ausscheidung der oben erwähnten Dämpfe, die aus exkrementellen Körpersäften entstehen. Die Ausdünstung selbiger findet nämlich zuallererst im Wachzustand statt. Nachdem also im vorliegenden Fall sowohl das Schlafen als auch das Wachsein positive Effekte hervorrufen, befürwortet Gallo ein ausgewogenes Verhältnis beider Zustände bei Maximilian.

Auch in anderen Lebensbereichen setzt Gallo – das war für die ärztliche Diätetik charakteristisch – auf Ausgewogenheit und Maßhalten: Maximilian ist während seiner Spanienreisen extremen Wetterbedingungen ausgesetzt gewesen, sehr kalter, aber auch sehr heißer Witterung, was sein Herz geschwächt haben dürfte. Das Verweilen bei gemäßigtem, heiterem Wetter stärkt hingegen das Herz, da es die Seele erfreut, und ist somit für Maximilian wesentlich besser geeignet. Zudem habe der übermäßige Beischlaf Maximilians mit seiner Ehefrau, die er im Jahr 1548 heiratete, sein Herz geschwächt [14]. Auch hier gilt es, unbedingt Maß zu halten. Weil sich heftige Emotionen wie große Freude oder starke Trauer negativ auswirken, empfiehlt Gallo, Tätigkeiten und Umstände zu bevorzugen, die mit moderater Freude verbunden sind. Auch von anstrengenden Tätigkeiten wie etwa dem Jagen oder dem Reiten solle sich Maximilian fernhalten. Hingegen beeinflussen maßvolle körperliche Ertüchtigungen in heiterer Atmosphäre die Beschwerden positiv.

Zusammenfassend lassen die Schilderungen in Gallos Konsil aus heutiger Sicht am ehesten vermuten, dass es sich bei Maximilians Herzzittern um ein primär psychosomatisches Geschehen handelt. Gallo selbst schreibt den Affekten und dem subjektiven Befinden Maximilians große Bedeutung für die Entstehung und Heilung der Krankheit zu. Seine Deutung des Krankheitsgeschehens als Folge einer *intemperies *lässt jedoch – typisch für jene Zeit – grundsätzlich eine dezidiert somatische Zugangsweise erkennen. Diese speist sich aus den damals weithin akzeptierten Vorstellungen über die Physiologie und Pathologie des Herzens und des *spiritus vitalis *und der Beeinträchtigung der Herzfunktion durch aufsteigende Dämpfe. Erst die präzise Rekonstruktion im Kontext der zeitgenössischen medizinischen Theorien, wie sie in diesem Beitrag beispielhaft unternommen wurde, kann die innere Logik von damaligen ärztlichen Deutungen und Empfehlungen erhellen und bewahrt so davor, Schlussfolgerungen, die im Einklang mit den herrschenden wissenschaftlichen Vorstellungen standen, als bloße Kuriosa abzutun.
